# Changes in the psychosocial and clinical profiles of anorexia nervosa patients during the pandemic

**DOI:** 10.3389/fpsyt.2023.1207526

**Published:** 2023-07-19

**Authors:** N. Burcu Özbaran, Hazal Yağmur Yılancıoğlu, Sibel Helin Tokmak, Begüm Yuluğ Taş, Didem Çek, Tezan Bildik

**Affiliations:** ^1^Department of Child and Adolescent Psychiatry, Faculty of Medicine, Ege University, İzmir, Türkiye; ^2^Çigli Education and Training Hospital, Bakırçay University, İzmir, Türkiye

**Keywords:** anorexia nervosa, COVID-19, pandemic, psychosocial, symptom change, eating disorder

## Abstract

The COVID-19 pandemic and related control measures have increased the prevalence of anorexia nervosa (AN), and recent studies demonstrated that approximately 70% of individuals diagnosed with AN experienced deterioration in symptoms. This study aimed to examine the psychosocial and clinical impacts on patients with AN during the COVID-19 pandemic. This cross-sectional study involved 35 female AN adolescents who were being treated at Ege University Child and Adolescent Psychiatry Department. To assess pre-pandemic symptom levels and daily life routines, a retrospective form utilizing the visual analog scale (VAS) was employed. Body mass index (BMI) and Clinical Global Impression (CGI) data were recorded by an interviewer for all 35 patients who gave consent to participate in the study. Fifteen patients completed all the forms [VAS, the Children's Depression Inventory (CDI), the Screen for Child Anxiety Related Disorders Scale (SCARED), the Eating Attitudes Test, the Quality-of-Life Scale (QoLS), the Autism Spectrum Screening Questionnaire, and the Turgay DSM-4 Based Screening and Evaluation Scale for Behavioral Disorders in Children and Adolescents] online in 2022. Clinical diagnosis and progress were assessed retrospectively using The Kiddie Schedule for Affective Disorders and Schizophrenia (K-SADS). The duration of the follow-up period and the number of psychiatric visits were obtained retrospectively from patient files. Retrospective data on pre-pandemic symptom levels and daily life routines were collected using the VAS. The mean age of AN patients was 16.67 ± 1.63 years. Comorbid diseases were present in 73.33% of patients, and major depressive disorder (MDD) was the most common (46.66%). Mean scores indicated moderate levels of anxiety (SCARED score: 37.23 ± 12.67) and depression (CDI score: 17.23 ± 10.85). QoLS scores were negatively correlated with eating attitudes (r = −0.601, *p* = 0.039). Obsession level, exercise level, and screen time increased during the pandemic, while social activity, quality of education, and perception of learning decreased. BMI increased in all patients. Patients who completed the forms had a higher number of psychiatric visits compared to those who did not complete the forms (*p* = 0.033). The mean number of clinic visits was 26.27 ± 20.33. The results show that the COVID-19 pandemic had adverse effects on AN patients and disrupted their daily routines. These patients experienced high rates of comorbidities. The patients showed improvement in BMI scores, indicating the positive impact of treatment. These findings emphasize the need for comprehensive psychiatric care and targeted interventions for AN patients during crises such as the COVID-19 pandemic.

## Introduction

Anorexia nervosa (AN) is a mental disorder characterized by a restriction of energy intake, fear of weight gain, distorted body image, and intense efforts to lose weight, leading to a significant reduction in body weight. Despite its critical significance, patients often struggle to comprehend their condition. Eating disorders typically emerge during adolescence and young adulthood (1). Although anorexia is more prevalent among adolescent females, its lifetime prevalence in women can reach up to 4% (2). The uncertainty caused by the physical and mental changes experienced in adolescence is believed to trigger anorexia by inducing a sense of loss of control (3). According to DSM-5, approximately 0.012% of patients with anorexia nervosa exhibit suicidal tendencies, and this rate is gradually increasing. Anorexia nervosa is a serious illness with potentially life-threatening consequences (4).

Numerous studies have shown that the COVID-19 pandemic had a negative impact on the mental health of children and adolescents. Women and adolescents were considered one of the more vulnerable groups in terms of mental health issues (5). Isolation measures implemented during the pandemic contributed to increased psychiatric problems by hindering children and adolescents from engaging with their peers (6). Given the significance of adolescence in terms of establishing peer relationships and individuation, the pandemic presented significant challenges to the mental wellbeing of young individuals (7). Limited social opportunities within the home environment led to increased social media exposure among young people, which is believed to contribute to the rise in mental illnesses among children and adolescents during the pandemic (8).

The incidence of eating disorders increased during the COVID-19 pandemic. The pandemic period was also associated with a higher incidence of anorexia nervosa and worsening symptoms of the disease. Studies showed that the number of newly diagnosed anorexia nervosa cases increased during this period (9). The need for hospitalization among individuals previously diagnosed with anorexia nervosa also increased (10). The increase in the disease was particularly prominent in adolescence and among women, with anorexia nervosa occurring at a rate 15.2% higher than in previous years. Suicidal thoughts and attempts were more prevalent among individuals with anorexia compared to previous years (11). Anorexia patients experienced emotional distress, anxiety, a sense of loss of control, and disrupted eating and sleeping patterns due to social interaction problems, decreased physical activity, extended periods spent at home, and uncertainty about the future during the pandemic (12, 13). These factors contribute to the development and exacerbation of anorexia symptoms. Recent studies have shown that approximately 70% of anorexia nervosa patients experienced worsened symptoms, such as disturbed body image and fear of weight gain, during the COVID-19 pandemic (14). Moreover, restrictive eating patterns and self-induced vomiting behaviors increased among patients during quarantine periods (15). Understanding the impact of the pandemic on symptoms and prognosis of anorexia nervosa patients can facilitate the development of interventions for eating disorders and guide the implementation of necessary precautions during future pandemics or long-term social restrictions (9).

In the existing literature, there is a scarcity of studies examining anorexia nervosa specifically during the COVID-19 pandemic (16). Moreover, there are no studies in Turkey investigating the alterations in the clinical presentation of anorexia nervosa during this challenging time. Therefore, the primary objective of the present study was to investigate the psychiatric and psychosocial effects, as well as the clinical changes, observed in patients diagnosed with anorexia nervosa who were under the care of the Ege University Child and Adolescent Psychiatry Clinic during the pandemic period. By addressing this research gap, we aimed to contribute valuable insights into the understanding of anorexia nervosa within the context of the COVID-19 pandemic.

## Methods

This study was conducted between June and August 2022. Anorexia nervosa patients who were being treated at the Eating Disorder Sub-Clinic of Ege University Child and Adolescent Consultation Liaison Psychiatry during the COVID-19 pandemic were contacted by phone. The inclusion criteria for participation in the study were clinically normal mental capacity, age between 13 and 18 years, voluntary agreement to participate, and reading and signing the voluntary consent form. The exclusion criteria included being younger than 13 years or older than 18 years, not having clinically normal mental capacity, unwillingness to participate, and the presence of concomitant neurological diseases.

Detailed information about the study was provided to 41 anorexia nervosa patients and their families. Six patients declined to participate, resulting in a final sample size of 35 anorexia nervosa patients. Written informed consent was obtained online from the patients and their families who agreed to participate.

This study followed a cross-sectional design. The patients were interviewed face-to-face once within the scope of the study, and the scales were completed online by the patients. The completed scales captured data from the pandemic period. To assess pre-pandemic symptom levels and daily life routines, a retrospective form utilizing the visual analog scale (VAS) was used during the pandemic period. A specific form was developed by researchers using the VAS, which ranged from 1 to 10, to investigate eating attitudes, patients' perceptions of daily routines (such as sleep, exercise, and technology use), and the level of clinical symptoms (such as anxiety and obsession) before and during the pandemic (17). In addition to the VAS form, other scales were completed by the participants online. These included the Children's Depression Inventory (CDI) to assess the severity of depression (18), the Screen for Child Anxiety-Related Disorders Scale (SCARED) to evaluate childhood anxiety disorders (19), the Eating Attitudes Test (EAT-26) to assess eating attitudes (20), and the Quality of Life Scale (QOLS) to measure the individuals' perception of their health and functioning in physical, psychological, social, and cognitive domains (21). The caregivers of the patients also completed the Autism Spectrum Screening Questionnaire (ASSQ) to assess social interaction, communication problems, restrictive and repetitive behaviors, motor clumsiness, and associated symptoms (22). Additionally, the Turgay DSM-4-Based Screening and Evaluation Scale for Behavioral Disorders in Children and Adolescents was used to evaluate attention deficit and hyperactivity/impulsivity in the children (23).

Psychiatric interviews were conducted using the Kiddie Schedule for Affective Disorders and Schizophrenia (K-SADS) to evaluate diagnoses and identify accompanying psychopathological conditions (24). The severity of the disease, treatment compliance, and level of clinical progress were recorded using the Clinical Global Impression (CGI) scale (25).

**Figure 1:**
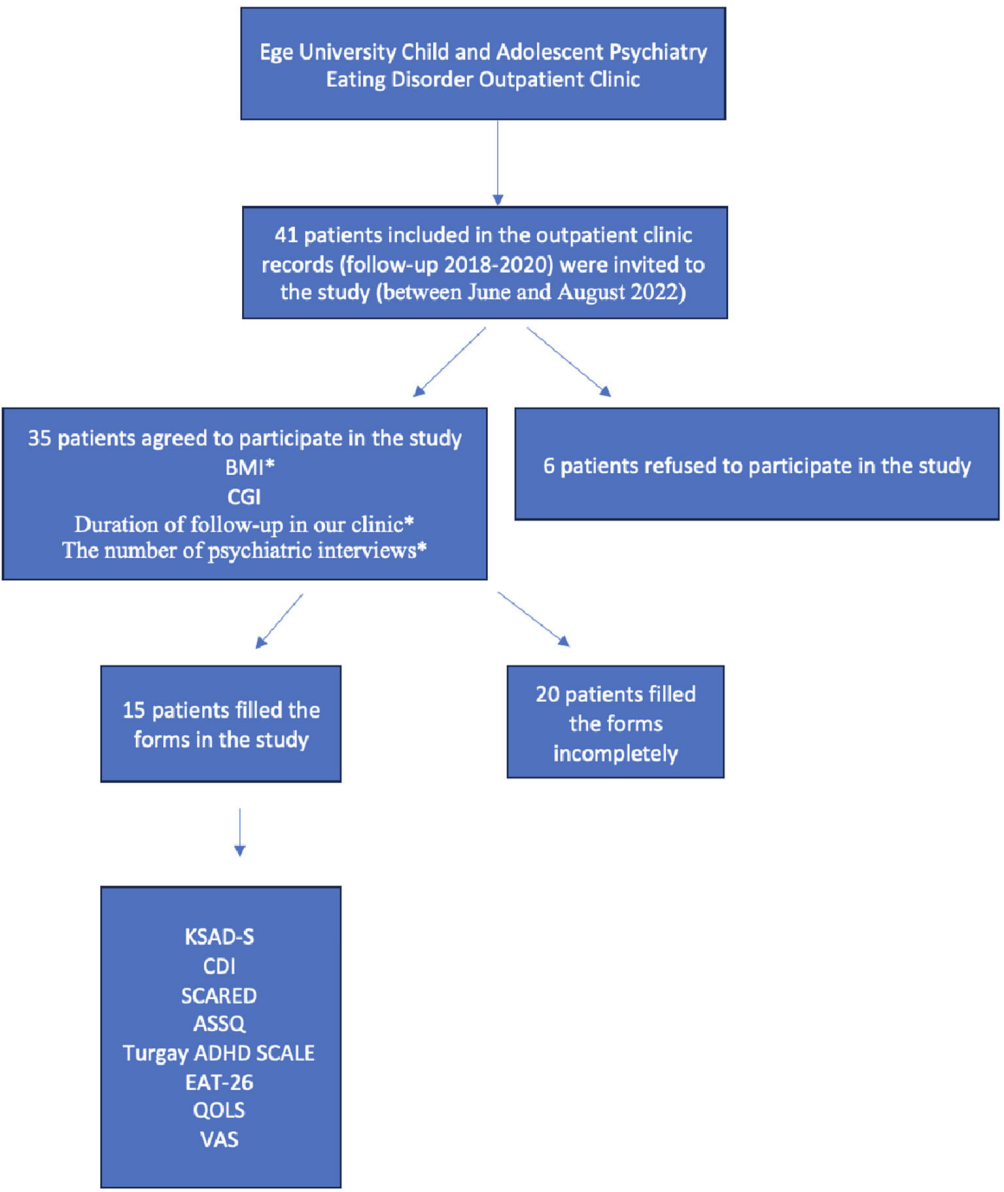
Representation of the steps followed in the study. *Data pertaining to the pre-pandemic body mass index (BMI) of patients, duration of clinic follow-up, and the number of psychiatric consultations were extracted from retrospective patient records.

A retrospective examination of the patients' files allowed for the retrieval of BMI data from their first visit to the clinic. BMI data obtained in the last interview during the pandemic were recorded as the last visit BMI. Furthermore, the duration of follow-up in the clinic and the number of psychiatric interviews were obtained from the files retrospectively and recorded for the 35 patients who participated in the study (Figure 1).

### Ethics approval

The presented study was conducted in accordance with the ethical standards laid down in the 1964 Declaration of Helsinki and approved by the local ethics committee. Written consent was taken from the patients and their parents. The study was approved by the Ege University Ethics Committee with decision number 22-6T/7.

### Statistical analysis

Descriptive statistics for quantitative variables were presented as mean ± standard deviation. Descriptive statistics for qualitative variables were expressed as frequency (%). The conformity of quantitative variables to normal distribution was assessed using the Shapiro–Wilk test. Pearson's correlation analysis was used to examine the relationship between normally distributed scales, while Spearman's correlation analysis was used for non-normally distributed variables. The Wilcoxon test was used to evaluate whether there was a significant difference between VAS measurements of daily routines before and during the pandemic. Categorical data for the pre-pandemic and pandemic periods were assessed using the McNemar test. The independent samples *t*-test was performed to compare the number of appointments, first-visit body mass indexes (BMIs), last-visit BMI, duration of follow-up in psychiatry, and clinical global impression scale scores between patients who participated and did not participate in the study. A *p*-value of < 0.05 was considered statistically significant.

## Results

Out of the initial sample of 41 patients, 6 declined to participate in the study. Thus, the study was conducted with a final sample size of 35 patients who provided voluntary consent. All 15 female adolescents with anorexia nervosa who participated in the study completed all the forms and questionnaires, including the K-SADS diagnostic interview. The mean age of the participants was 16.67 ± 1.63 years. According to the K-SADS diagnostic interview, 11 patients (73.33%) had at least 1 mental illness accompanying an eating disorder. Specifically, major depressive disorder (MDD) was diagnosed in seven patients (46.66%), anxiety disorder in three patients (20.00%), attention deficit and hyperactivity disorder in two patients (13.33%), and mood disorder in one patient (6.66%). The patients had a mean SCARED score of 37.23 ± 12.67, indicating anxiety symptoms, and a mean CDI score of 17.23 ± 10.85, indicating depressive symptoms.

Using the VAS, it was found that during the pandemic period, compared to the pre-pandemic period, there was a significant increase in the obsession level (z = −2.254, *p* = 0.024), exercise level (z = −2.508, *p* = 0.012), screen time (z = −2.290, *p* = 0.022), and time spent using connected technology (z = −2.373, *p* = 0.018). Conversely, the level of social activity (z = −2.206, *p* = 0.027), quality of education (z = −2.167, *p* = 0.030), and perception of learning (z = −3.301, *p* = 0.008) significantly decreased during the pandemic period. However, no significant difference was observed in anxiety levels (z = −0.891, *p* = 0.373) between the two time periods. No significant findings were found regarding self-harming behaviors, regular dietician applications, changes in menstrual patterns, or diuretic-laxative use during the pandemic compared to those before the pandemic. Similarly, there was no significant difference in activities such as hair coloring, reading manga, starting a new hobby, being interested in art, and playing a new instrument between the pre-pandemic and pandemic periods.

The patients had a mean ASSQ score of 6.93 ± 5.88, indicating autism spectrum traits, and a mean ADHD scale attention deficit level of 6.80 ± 5.33. A moderate positive correlation was found between the ASSQ and attention deficit scores of the patients (r = 0.668, *p* = 0.006). The mean total score on the quality-of-life scale was 64.80 ± 19.67, while the mean eating attitude scale score was 38.50 ± 23.45. There was a moderate negative relationship between quality-of-life scores and eating attitudes scores (r = −0.601, *p* = 0.039).

The mean number of visits for patients during the pandemic period in Turkey (2020–2022) was 26.27 ± 20.33. It was observed that patients who completed the forms (completers, *n* = 15) had a higher number of visits from the first appointments compared to patients who did not complete the forms (non-completers, *n* = 20) (*p* = 0.033). However, there was no significant difference between completers and non-completers in factors predicting recoveries, such as first-visit BMI, last-visit BMI, duration of follow-up in psychiatry, and clinical global impression scale (CGI) scores. The severity of the disease, as measured by the CGI at the first visit, was 5.33 ± 1.11, while the mean CGI improvement score recorded at the last interview was 2.93 ± 1.03.

Regarding BMI scores, there was a significant increase for all patients during the pandemic period (z = −3.301, *p* = 0.001 for completers; z = −3.384, *p* = 0.002 for non-completers).

## Discussion

The primary findings of our study highlight the psychiatric, psychosocial, and clinical impacts experienced by individuals with anorexia nervosa, providing evidence for the psychopathological effects of the COVID-19 pandemic. The results revealed that patients with anorexia nervosa exhibited increased screen time, higher levels of exercise, reduced participation in social activities, and decreased perceived learning levels during the pandemic. While the anxiety levels of the patients remained unchanged, there was an increase in obsessive symptoms and a decline in the quality of education due to social isolation and disrupted schooling. No significant changes were observed in self-destructive behaviors during the pandemic period (Table 1). Furthermore, our study identified an association between quality of life and eating attitudes during the pandemic period. It was also noted that patients with anorexia nervosa had a high comorbidity rate, indicating the presence of additional concurrent conditions. Despite the evolving symptom profile, an increase in patients' body mass index (BMI) and a decrease in the clinical severity of the illness were observed (Table 2). In summary, our research contributes to the understanding of the psychiatric, psychosocial, and clinical implications of anorexia nervosa during the COVID-19 pandemic. The observed changes in behaviors, perceptions, and comorbidity rates emphasize the need for tailored interventions and support for individuals with anorexia nervosa in the context of extraordinary circumstances. These findings have implications for future research and treatment strategies aimed at improving outcomes for individuals with anorexia nervosa.

**Table 1:** VAS scores before and during the pandemic period.

	**VAS scores before the pandemic (*n =* 15)**	**VAS scores during the pandemic (*n =* 15)**		
	**Mean** ±**SD**	**Mean** ±**SD**	**z**	***p***
Satisfaction with body image	5.13 ± 3.60	3.53 ± 1.00	−0.892	0.372
Family communication quality	2.84 ± 1.00	3.02 ± 1.00	−0.966	0.334
Number of meals per day	1.73 ± 1.98	1.33 ± 1.29	−0.849	0.396
Calorie counting	3.80 ± 4.29	5.27 ± 4.04	−1.012	0.311
Fear of weight gain	6.33 ± 3.99	6.87 ± 3.88	−0.284	0.777
Guilt after eating	4.93 ± 3.36	6.20 ± 3.44	−1.091	0.275
Vomiting	2.07 ± 3.08	3.07 ± 2.81	−1.809	0.070
Obsession level	3.60 ± 3.00	5.33 ± 5.00	−2.254	0.024^*^
Anxiety level	4.80 ± 3.12	5.60 ± 3.46	−0.891	0.373
Nutritional quality	3.60 ± 2.92	5.33 ± 3.57	−0.059	0.953
Sleep quality	6.33 ± 2.09	6.26 ± 2.93	−0.158	0.875
Social activity	5.33 ± 2.82	3.67 ± 2.63	−2.206	0.027^*^
Exercise	4.73 ± 2.71	6.93 ± 3.30	−2.508	0.012^*^
Domestic responsibilities	5.53 ± 2.53	6.53 ± 2.89	−1.827	0.068
Screen time (hour)	4.80 ± 2.30	6.00 ± 2.67	−2.290	0.022^*^
Technology use (hour)	3.86 ± 2.13	5.53 ± 2.85	−2.373	0.018^*^
Education quality	7.66 ± 2.66	5.53 ± 3.24	−2.167	0.030^*^
Learning states level	7.66 ± 2.69	4.66 ± 3.65	−3.301	0.008^*^

VAS, visual analog scale; ^*^p < 0.05, z, Wilcoxon test statistics.

**Table 2:** Clinical follow-up characteristics.

	**Completers (*n =* 15)**	**Non-completers (*n =* 20)**		
	**Mean** ±**SD**	**Mean** ±**SD**	**t**	***P***
First visit BMI	15.06 ± 2.01	15.80 ± 1.88	1.107	0.276
Last visit BMI	20.13 ± 3.37	18.55 ± 3.23	−1.406	0.169
CGI	5.33 ± 1.11	5.15 ± 0.93	−0.199	0.843
Follow-up time (month)	14.60 ± 11.90	8.80 ± 8.63	−1.673	0.104
Number of visits	26.27 ± 20.33	13.95 ± 12.39	−2.220	0.033^*^

BMI, body mass index, ^*^ *p* < 0.05, t, independent *t*-test.

Early studies conducted during the COVID-19 pandemic indicated a higher risk of symptom reactivation among individuals with pre-existing mental disorders (26). Reports showed an intensification of symptoms in individuals with anorexia nervosa during the pandemic (10, 13). Furthermore, evidence suggests that changes in routines can influence eating attitudes by altering eating patterns (27). A study conducted in Sweden examined the behaviors of individuals with anorexia during the COVID-19 pandemic, revealing increased social media use, exercise levels, and dietary restrictions. The increase in social media usage was identified as a significant contributing factor to the worsening of symptoms. This increase may be associated with disrupted eating patterns due to prolonged periods of staying at home and heightened body concerns among the patients (8). In an Australian study comparing individuals with and without a history of eating disorders, it was observed that those with eating disorders increased their exercise levels during the pandemic, while a decrease was observed in the general population. Specifically, approximately half of the patients in the anorexia subgroup exhibited an increase in exercise behavior (28). A study investigating factors contributing to the exacerbation of anorexia symptoms during the COVID-19 pandemic suggested that increased media exposure may worsen anorexia by exposing individuals to content related to body perception (16). In this context, the results of the present study regarding exercise and social media usage evaluated before and during the pandemic are consistent with the literature. However, there is a lack of sufficient research in the literature concerning other aspects such as the decrease in learning levels and perceived educational quality among anorexia patients with respect to online education during the pandemic.

Several studies noted a high prevalence of comorbid psychiatric disorders among patients with anorexia nervosa (29). Specifically, MDD was found to be present in approximately half of these patients. Consistent with our findings, MDD was consistently identified by previous studies as one of the most frequently observed comorbidities in individuals with anorexia nervosa (30). The COVID-19 pandemic has been associated with increased levels of depression and anxiety, largely attributed to the impact of traumatic events and mandatory isolation measures (31, 32). Notably, a study examining individuals with a history of anorexia reported that 50% of the participants experienced moderate to high levels of depression and anxiety symptoms during the COVID-19 period (28). In line with existing literature, our results also showed elevated levels of anxiety and depression among the participants.

It is well-established that anorexia nervosa can be associated with autistic features, such as difficulties in social cognition, emotion regulation, and abnormal attention processing (4). Previous studies have reported that female adolescents diagnosed with anorexia nervosa exhibit lower social communication skills and higher autistic characteristics (33). Approximately one-fifth of individuals with anorexia nervosa are reported to display autism-related features, which can manifest as challenges in communication, social analysis, and theory of mind (34). In the present study, we assessed the autism-related characteristics of patients using the Autism Spectrum Screening Questionnaire (ASSQ), highlighting the importance of considering these features during clinical follow-up. Attention-deficit/hyperactivity disorder (ADHD) is another neurodevelopmental disorder that commonly co-occurs with anorexia nervosa and can contribute to social skill impairments (34). These factors are believed to play a role in both the etiology and maintenance of the disease (34). Given the social restrictions imposed by the COVID-19 pandemic, it is possible that attention deficit and autistic features accompanying anorexia nervosa may further exacerbate the current situation.

Earlier studies have demonstrated that patients with anorexia nervosa exhibit low cooperation in psychiatric follow-ups, leading to frequent dropouts (35). Anorexia nervosa is known to be a resistant disease, and the clinical course can vary depending on patients' adherence to treatment. A meta-analysis examining the relationship between therapeutic compliance and clinical course in anorexia nervosa indicated that good treatment cooperation could positively influence treatment response, particularly among adolescents (36). In our study, we found a strong positive correlation between the duration of clinical follow-up and the final BMI. Furthermore, we observed that all patients, including both completers and non-completers of the clinical follow-up, experienced an increase in their BMI during the study period (Table 3).

**Table 3:** BMI change between the first and last visit.

	**First visit BMI**	**Last visit BMI**		
	**Mean** ±**SD**	**Mean** ±**SD**	**z**	***p***
Completers (*n =* 15)	15.06 ± 2.01	20.13 ± 3.37	−3.301	0.001
Non-completers (*n =* 15)	15.80 ± 1.88	18.55 ± 3.23	−3.584	0.002

BMI, body mass index; z, Wilcoxon test statistics.

## Limitations

The limited sample size in our study imposes constraints on the generalizability of the findings. It was observed that patients with anorexia nervosa encountered difficulties in completing the forms due to their negative attitudes. Furthermore, our study exclusively consisted of female participants, which prevented us from conducting gender-specific analyses. To better capture the impact of changing disease symptoms, it was deemed valuable to examine different phases of the pandemic, such as the early and later stages, and to evaluate the effects of acute stressors. It is important to acknowledge the potential for recall bias since the patients' pre-pandemic symptoms were assessed during the interview conducted during the pandemic period.

## Conclusion and suggestions

In conclusion, the results obtained in this study shed light on the heightened symptomatology of anorexia nervosa observed during the COVID-19 pandemic. The sharing of clinical experiences and insights gained from the mental health of our patients during this unprecedented period holds the potential to contribute to the development of tailored treatment approaches and the effective management of similar extraordinary situations that may arise in future. Therefore, it is crucial to continue exchanging clinical experiences and disseminating findings from follow-up studies in order to enhance our understanding and improve treatment outcomes for individuals diagnosed with anorexia nervosa. By building upon this collective knowledge, we can optimize interventions and support strategies to better address the unique challenges faced by individuals with anorexia nervosa in times of crisis and beyond.

## Data availability statement

The raw data supporting the conclusions of this article will be made available by the authors, without undue reservation.

## Ethics statement

This study was reviewed and approved by the Ege University Ethics Committee and its approval number is 22-6T/7. Written informed consent was obtained online from patients and families who agreed to participate in the study.

## Author contributions

NÖ, HY, ST, BY, and DÇ: study design and method and data collection. HY: analysis of data. NÖ, HY, BY, and ST: preparation of the original draft. NÖ and TB: review. All authors contributed to the article and approved the submitted version.
